# Heat Wave Intensity Drives Sublethal Reproductive Costs in a Tidepool Copepod

**DOI:** 10.1093/iob/obac005

**Published:** 2022-01-31

**Authors:** Matthew R Siegle, Eric B Taylor, Mary I O'Connor

**Affiliations:** Department of Zoology and Biodiversity Research Centre, University of British Columbia, V6T 1Z4 Vancouver BC, Canada; Department of Zoology, Biodiversity Research Centre and Beaty Biodiversity Museum, University of British Columbia, V6T 1Z4 Vancouver BC, Canada; Department of Zoology and Biodiversity Research Centre, University of British Columbia, V6T 1Z4 Vancouver BC, Canada

## Abstract

Physiological stress may induce sublethal effects on fitness by limiting energy availability and shifting energy allocation, which can incur reproductive costs. Sublethal reproductive costs may affect vital rates, linking stress events such as heat waves to population demography. Here, we test the hypothesis that heat wave intensity and consecutive days of exposure to heat wave temperatures impact survival and individual reproductive success. We subjected groups of the marine harpacticoid copepod, *Tigriopus californicus*, to 6 heat wave regimes that differed in maximum exposure temperature, 26°C or 32°C, and number of consecutive exposure days (1, 2, or 7), and predicted that survival and reproductive costs would increase with heat wave intensity and duration. We measured individual survival and offspring production during the heat waves and for 2 weeks following the last day of each experimental heat wave. Despite similar survivorship between the 2 maximum temperature treatments, sublethal effects of heat wave intensity were observed. Consistent with our predictions, individuals that experienced the higher maximum temperature 32°C heat waves produced fewer offspring overall than those that experienced the 26°C heat wave. Furthermore, the number of naupliar larvae (nauplii) per clutch was lower in the 32°C group for egg clutches produced immediately after the final day of exposure. Our results are consistent with the hypothesis that increasing thermal stress can lead to sublethal costs, even with no discernible effects on mortality. Heat waves may not always have lethal effects on individuals, especially for individuals that are adapted to routine exposures to high temperatures, such as those occupying the high intertidal. Costs, however, associated with stress and/or reduced performance due to non-linearities, can affect short-term demographic rates. The effect of these short-term sublethal perturbations is needed to fully understand the potential for population rescue and evolution in the face of rapid climate change.

## Introduction

Temperature is one of the most fundamental abiotic variables affecting animal performance, especially in ectotherms (Huey 1982; Angilletta et al. 2002; Hochachka and Somero 2002; Trudgill et al. 2005; Schulte 2015). As climate change accelerates, understanding the biological responses to extreme temperature events has never been more pressing. Climate models predict a warmer world that includes an increase in the frequency, duration, and severity of temperature events, or heat waves (Frich et al. 2002; Meehl and Tebaldi 2004; Rahmstorf and Coumou 2011). Thermal variability affects both population and individual growth rates differently than changes in mean temperatures (Bozinovic et al. 2011; Folguera et al. 2011; Estay et al. 2013; Denny 2017; Morash et al. 2018). Population growth rates and individual growth and energy allocation are important components of fitness, and therefore affect the chance of a population persisting in a changing world (Vasseur et al. 2014; Buckley and Huey 2016).

Heat waves may impact fitness through lethal and sublethal components. Exposure to high temperatures may result in large-scale mortality events (Tsuchiya 1983; Garrabou et al. 2009; McKechnie and Wolf 2010), but may also reduce growth and/or reproduction. Survival and reproductive costs are likely mediated by constraints on energy production and allocation. Under a period of heat stress, energy normally allocated to growth and reproduction under non-stressful conditions may be re-allocated to the maintenance of energetic balance (homeostasis) in response to increasing thermal stress (Somero 2002; Helmuth et al. 2010; Sokolova et al. 2012; Sokolova 2013).

Compounding the effects of heat waves on energy allocation, extreme heat events may further exacerbate the effect of temperature on energy balance through changes in energy production and reductions in total energy availability. At higher temperatures, aerobic scope, that is, the total energy available beyond maintaining basal metabolic functions, declines as oxygen concentration decreases (Pörtner and Knust 2007). As oxygen concentration decreases, energy production shifts away from higher yielding aerobic pathways to lower yielding anaerobic ones resulting in lower total energy production (Verberk and Calosi 2012). Additionally, energetically expensive physiological stress responses may be activated in response to cellular damage (Bell et al. 1988; Feder and Hofmann 1999; Bragg and Wagner 2009; Hoekstra and Montooth 2013). These concurrent changes in energy allocation, energy production and energy availability extend an individual's survival time in stressful conditions. These changes in individual physiology, however, can impair individual fitness, and therefore its contribution to population persistence over the longer term, by reducing reproductive effort. Advancing frameworks that link models of energy and temperature (Pörtner 2010; Pörtner 2012) to ecological models of energy budgets (Kooijman 2010) and fitness is critical for mechanistically understanding the sublethal effects of physiological stress to ecologically relevant demographic rates (Sokolova et al. 2012; Sokolova 2013).

Individual fitness links physiological processes to the population vital rates that underlie population persistence in the face of environmental stress. The impact to individual fitness, however, depends upon on the type of the stressor, the intensity of the stressor, and the duration of the exposure period (Rohmer et al. 2004; McNamara and Buchanan 2005). The effects of temperature stress on individual survival and reproductive effort may be hard to predict, due to the non-linearity with which many metabolic processes scale with temperature. Furthermore, thermotolerance is plastic, and resistance to stressful temperatures reflects both long-term evolutionary adaptation and short-term effects of thermal history (Angilleta 2009; Schulte 2015). Moreover, while the physiological responses to heat stress such as the heat shock response are well studied (Lindquist and Craig 1988; Schlesinger 1990; Hightower 1991), organismal responses may not scale linearly with repeated heat stress events (Smith 2011), and may instead change rapidly, leading to population level-effects that exceed a threshold and have long-lasting effects on population demography (Harley and Paine 2009).

The objectives of this study were to test the hypothesis that lethal and sublethal costs increase with increasing heat wave intensity and duration. We tested for these heat wave effects in the splash pool copepod, *Tigriopus californicus* (Copepoda: Harpacticidae). We subjected individuals to one of six experimental heat wave regimes varying in heat wave intensity (daily maximum temperature: 26°C or 32°C) and exposure duration (consecutive days of exposure were 1, 2, or 7). We tracked survival, egg clutch production, and offspring production to estimate individual fitness.

## Methods

### Study system, site description, and field collection

The splash pool copepod, *T. californicus*, is an organism well-suited for investigations of the effect of heat stress on energy balance and fitness. This copepod species is distributed from Baja California to southeastern Alaska in the northeastern Pacific Ocean. Populations occupy splash pools in the supralittoral zone of the high intertidal—a zone characterized by daily temperature fluctuations and highly variable salinity conditions. The replenishment of splash pools with seawater may only occur during the highest tides each tidal cycle or with storm events, 1 or 2 days per month (Egloff 1966; Dybdahl 1994).

The reproductive life history of *T. californicus* makes it highly amenable to experimental manipulation and quantification of fitness metrics. Over approximately 3–4 weeks individuals develop through 12 life stages: six naupliar stages (N1–N6) and five copepodid stages (C1–C5) to reach the adult stage. Adult males clasp immature females, and immediately after a female's final molt into adulthood, a male will inseminate and then release the female. Males may mate more than once, but females generally mate only once. However, females are capable of producing multiple broods from the same insemination even (Burton 1985). Eggs brood on the ventral side of the urosome where the egg clutches are readily apparent. Females typically produce around 30 nauplii per clutch (Powlik et al. 1997). The clasping behavior of mating pairs, visibility of gravid females, and moderate number of nauplii produced per clutch make tracking reproductive effort relatively straightforward.

We collected individuals from splash pools located within the rocky shores of Botany Bay. 48°31.7’N, 124° 27.1’W. in Juan de Fuca Provincial Park, located on southwestern Vancouver Island near Port Renfrew, BC (Supplementary Fig. 1). Botany Bay is located at the westernmost entrance to the Strait of Juan de Fuca and is characterized by cool maritime weather due to fog, cloud cover, and high precipitation (annual mean precipitation is around 2.5 m). Summer air temperatures range from a mean low of 12°C to a mean high of 23°C. Individuals were collected in November 2015. Collections were composed of individuals from approximately five neighboring splash pools, that were brought back to The University of British Columbia, where they were combined and housed in 500 mL jars and 10 L aquaria in November and May. Water temperature in the lab ranged from 19–21°C, consistent with small fluctuations in room temperature, and salinity was maintained at 30–34 ppt. *Tigriopus* cultures were fed ground *Spirulina* algae (Max Pro brand fish food) *ad libitum*.

### Heat wave experiments

**Fig. 1:**
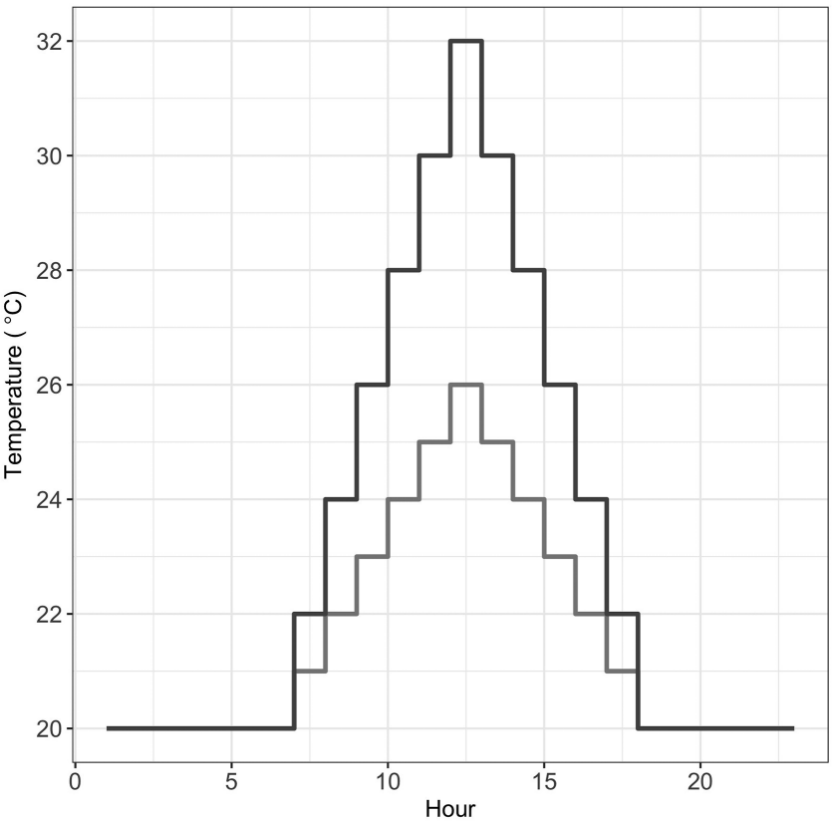
Daily temperature regime for the experimental heat waves to which the groups of *T. californicus* copepods were exposed. The black line is for the 32°C heat waves and grey line is for the 26°C heat waves.

To test our hypothesis, we subjected groups of copepods to one of six experimental heat wave scenarios. The simulated heat waves reflect both realistic daily temperature fluctuations as well as realistic heat wave durations (Siegle et al. 2018). Daily fluctuations of experimental heat waves included a six-hour gradual increase from the minimum temperature (20°C), one hour at the maximum temperature (26°C or 32°C), and a six-hour decline back to the minimum temperature (Fig. 1). The six heat wave treatments included heat wave durations of 1, 2, or 7 consecutive days of exposure at either maximum temperature. The maximum temperatures were selected to span a range of high temperatures encountered in the field. Field summer temperature data show that 26°C is in the 50^th^ percentile of daily maximum temperature and 32°C is in the 5^th^ percentile of daily maximum temperature (Supplementary Table 1, Supplementary Fig. 2). We recorded summer splash pool hourly temperatures by epoxying ibutton data loggers (Embedded Data Systems) into 6–12 splash pools during the summers of 2014 and 2015. The thermal environments in the lab were created with a Panasonic M1R-154 programmable incubator.

We tested the hypothesis that increasing heat wave intensity and duration lead to increasing costs to individual fitness. We subjected mated females to heat wave treatments and tracked female survival and reproduction for 20 days following the first day of the experimental heat waves. We isolated mate-clasping pairs in 6 mL wells of a 12-well plate. Each well was filled with 4–5 mL of sterilized seawater (28–32 ppt). By isolating clasping pairs, we ensured that we tracked females at the beginning of their reproductive life-stage (Burton 1985), rather than females that had already produced multiple broods or were post-reproductive. Pairs were held at 20°C for 12 days, then subjected to the heat wave treatments. The sample size of clasping pairs was 29–32 for each heat wave treatment. After each heat wave, individuals were held at 20°C with a 16:8 hour light:dark cycle for the remainder of the experiment. Each pair received ∼0.05 mg of ground *Spirulina* algae (Nutrifin Max brand) at day 1, 4, 8, and 16 of the experiment. This addition, in conjunction with some natural algae growth provided ample food without excessive bacterial growth. We periodically conducted 50% water changes and added distilled water to maintain salinity. Feeding and water changes occurred on the same day for all treatments.

To reduce the possibility of cannibalism (Gallucci and Ólafsson 2007), males were removed and discarded when the first egg clutch appeared. After nauplii hatched or egg sacs degraded, the female was transferred to a new well. This process was repeated for each subsequent egg clutch. We checked each individual daily for clasping status (still clasped vs. unclasped), appearance of an egg clutch, mortality, and nauplii hatching. Nauplii were collected two to three days after hatching and preserved in 75% ethanol. Egg clutches that had not hatched by the end of the experiment were not used in any subsequent analyses on the rate of nauplii production. A Leica M165C stereoscope was used to count the number of nauplii produced from each egg clutch.

### Statistical analyses

1. Fitness assays–Female survival

We estimated female survival probabilities during the experimental heat waves and for 2-weeks post heat wave using the standard non-parametric Kaplan–Meier (KM) survivorship function (with right-censored data) (Kaplan and Meier 1958). The probability of survival, *S*, depends upon the number of deaths divided by the number at risk. (the hazard function; λ, at a particular time point, *t*). The KM method updates the information at each censoring event, i.e., each death).

$$\begin{equation*}S(t = S\left( {t - 1^*1 - \lambda \left( t \right)} \right).\end{equation*}$$

We first used the Gehan–Breslow (modified Wilcoxon) test to test for differences in the survival curves between the 26°C and 32°C groups. This test weights earlier time points, and because the heat waves occurred at the beginning of the experiment we expected most mortality to occur during this time. Next, we performed a Log-rank test, giving equal weight to all time points, to test for differences in the survival curves between the two temperature groups. The Gehan–Breslow and Log-rank tests were performed with the *survMisc* package in R (Dardis 2015).

2 Fitness assays–Female reproduction

Total lifetime reproductive output can be impacted by reducing the number of clutches a female produces, and by reducing the number of offspring per clutch while reproductive. To distinguish between the effects on total reproduction and the immediate effects on offspring per clutch, we standardized the total number of nauplii produced by a female by the total number of days she persisted in the experiment. Consequently, we only measured the direct of effect of heat wave intensity and duration on short-term reproductive output and not on total lifetime reproduction. We performed a two-way ANOVA to investigate the effect of heat wave intensity and duration on the standardized number of offspring produced per female. We performed a Wilcoxon Rank Sum test in the *coin* package (Hothorn et al. 2006, 2008) in R to test for an effect of temperature intensity on the number of nauplii per clutch. To investigate the acute effects of heat stress on reproduction, we also restricted the analysis of temperature intensity on the number of nauplii per clutch to only those clutches produced immediately after the last day of the heat wave.

We also investigated the time to egg clutch production using the Kaplan–Meier method described above for the survival analysis.

## Results

**Fig. 2:**
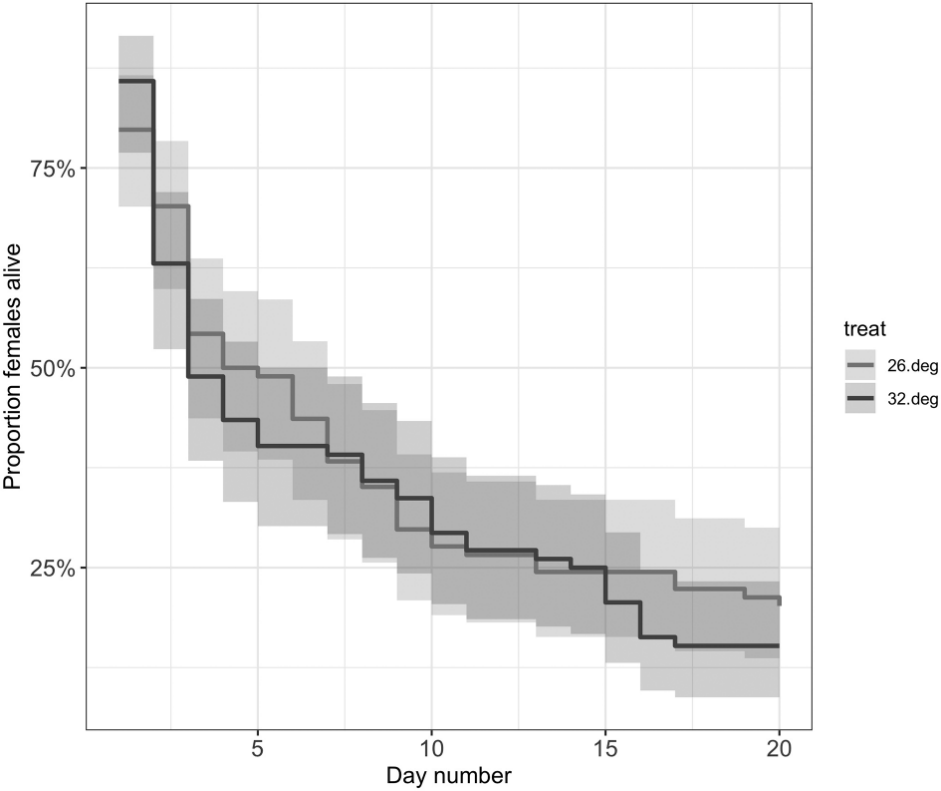
Estimated daily probability of *T. californicus* survival for the 26 and 32°C heat wave intensity groups. Data are pooled across all exposure duration levels. No difference in survival probabilities (+95% CI) was observed between the two temperature groups.

Survivorship did not differ between the two temperature intensity groups (Fig. 2; Log-rank test: χ^2^ = 0.385, df = 1, *P* = 0.53 and Gehan–Breslow test: χ ^2^ = 0.165, df = 1, *P* = 0.68). Mortality rates were highest in the first 2–3 days of the heat waves, and declined thereafter.

**Fig. 3:**
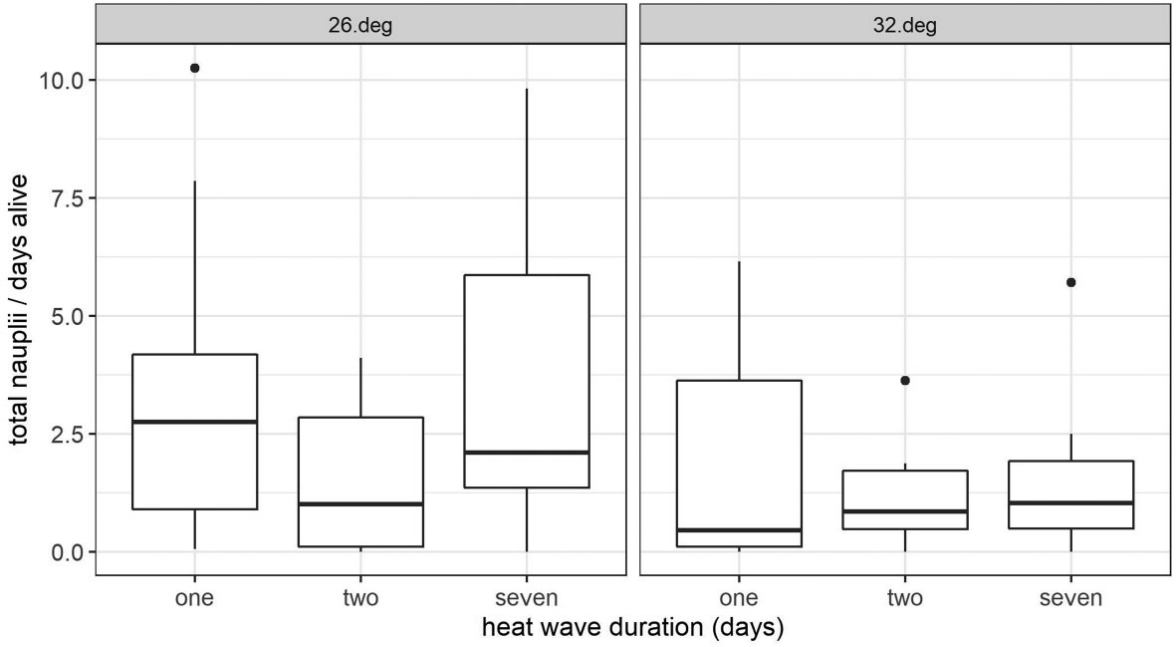
Total nauplii produced per female standardized by the number of days each female lived during the experiment. Overall offspring production decreases with increasing temperature. This effect is not observed, however, within the 26°C/2-day duration group. Boxplots show median values (horizontal lines) and 1^st^ and 3^rd^ quartiles (upper and lower edges of boxes). Whiskers encompass all values that are +/− 1.5 times the distance between the 1^st^ and 3^rd^ quartiles. Outliers are shown as dots.

We measured an additional two components of fitness; the number of egg clutches produced as well as the total number of nauplii that hatched. A total of 116 egg clutches containing 2199 nauplii were produced. Zero to 75 nauplii were produced per clutch, while 9.6% (7 out of 73) and 18.6% (8 out of 43) of egg clutches yielded no nauplii in the 26°C and 32°C groups, respectively. Per capita total reproductive output, estimated as the number of offspring, declined in the high-intensity 32°C group compared to the low intensity group (t = −2.313, df = 61, *P* = 0.024) (Fig. 3).

**Fig. 4:**
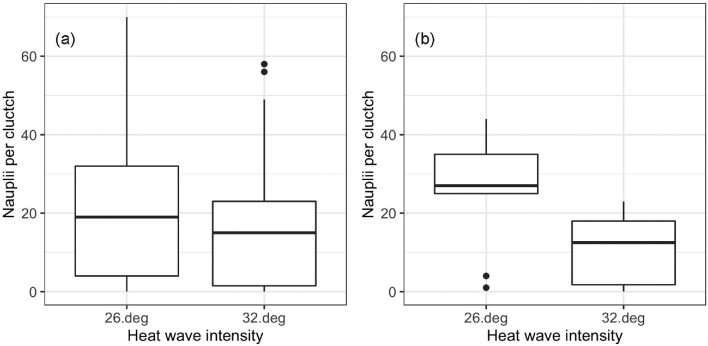
Number of *T. californicus* nauplii per clutch. The number of nauplii per clutch for each temperature intensity group is shown for all 116 clutches over the entirety of the experiment (a), and for the 15 clutches produced immediately after the last day of exposure (*n* = 9 and 6 for the 26 and 32°C groups, respectively) (b). No significant difference was found when considering all 116 clutches. Increasing temperature, however, reduced the number of nauplii per clutch for the restricted 15 egg clutches. Boxplots show median values (horizontal lines) and 1^st^ and 3^rd^ quartiles (upper and lower edges of boxes). Whiskers encompass all values that are +/− 1.5 times the distance between the 1^st^ and 3^rd^ quartiles. Outliers are shown as dots.

**Fig. 5:**
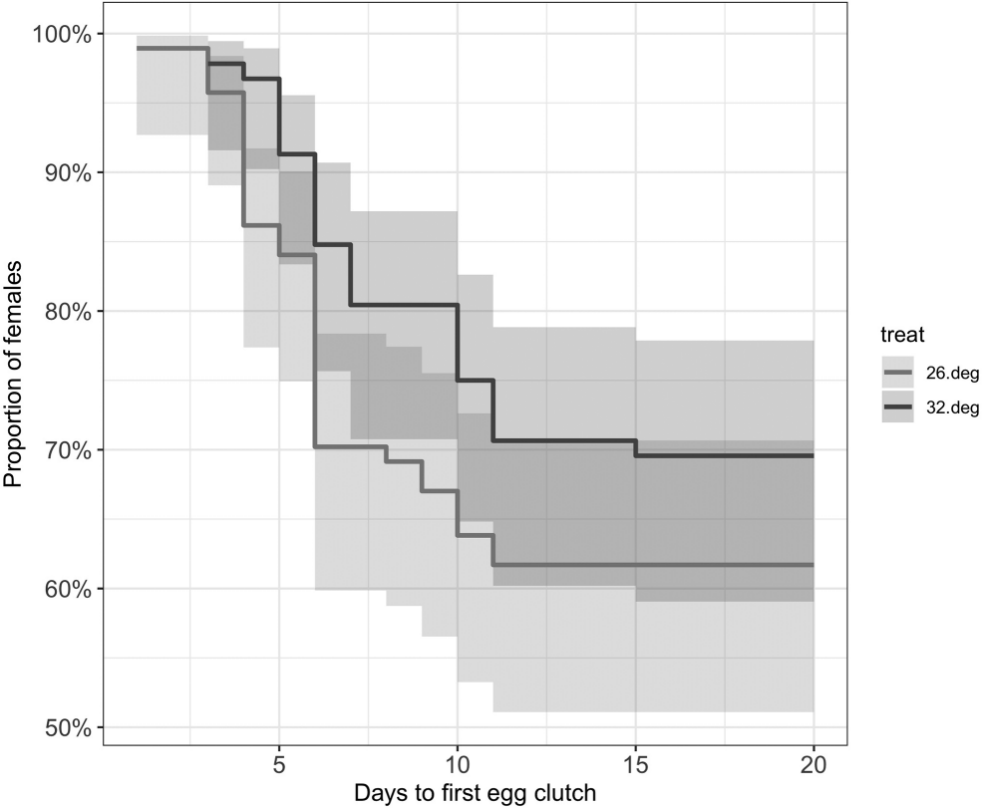
Probability of time to first egg clutch for *T. californicus* females. The proportion of individuals that have produced their first egg clutch at each time step are shown for both the 26 and 32°C groups, pooled across all exposure duration levels. No statistical difference was found between the two temperature groups, however, there was a trend towards the higher temperature group delaying production of their first egg clutch (Log-rank test: χ^2^ = 1.84, *P* = 0.18). The lines represent the estimated probabilities and the shaded regions represent the 95% CIs.

We did not detect an effect of heat wave intensity on the number of nauplii per clutch across all 116 clutches (mean nauplii per clutch: 21.01 and 15.47 for the 26°C and 32°C groups, respectively; Z = 1.63, *P* = 0.103) (Fig. 4A). By contrast, when we restricted the analysis to the subset of egg clutches that were produced immediately after the last day of the heat wave, and excluded those produced two or more days after the temperature returned to a constant 20°C (*n* = 9 and 6 clutches for the 26°C and 32°C groups, respectively), increasing temperature intensity reduced the number of nauplii per clutch (mean nauplii per clutch: 26.1 and 11.0 for the 26°C and 32°C groups, respectively; Z = 2.24, *P* = 0.025) (Fig. 4B). We observed a slight trend of increasing heat wave duration negatively affecting offspring production, despite not being statistically significant (Day 2: t = −1.90, df = 59, *P* = 0.063; Day 7: t = 0.33, df = 59, *P* = 0.74). We found that the time to first egg clutch production was delayed for the 32°C heat wave intensity group; however, this slight trend was not statistically significant (Log-rank test: χ^2^ = 1.84, *P* = 0.18) (Fig. 5).

## Discussion

We tested the hypothesis that increasing heat wave intensity and heat wave duration would increase lethal and sublethal fitness costs. We found that survivorship did not differ between individuals in the different temperature intensity groups. Females in the high intensity 32°C heat wave group, however, produced fewer overall offspring than those in the 26°C heat wave group, and the number of nauplii per clutch was lower in the 32°C group than the 26°C group for clutches produced immediately after the last day of heat wave exposure. Thus, survivorship in the 32°C group was maintained at a similar rate as the 26°C group, but individuals exhibited reduced per capita reproductive output. These results are consistent with the hypothesis that increasing thermal stress may increase sublethal costs despite similar patterns of mortality.

### Effects on fitness

As predicted, offspring production decreased with increasing temperature intensity. Several factors possibly contributed to this result: a decrease in the number of eggs produced by females in the 32°C treatment, differential mortality rates of N1–N2 nauplii, a decline in hatching success, and a delay in egg sac production. We found that females in the 32°C treatment slightly delayed egg sac production relative to females in the 26°C treatment, however, these differences were not statistically significant. We let the nauplii grow for two to three days post hatching to attain a large enough size to visualize and count effectively under the microscope. As such, we are not able to differentiate the contributions of a decline in egg number, a decline in hatching success, and a disproportionately higher mortality rate of N1–N2 nauplii in the 32°C treatment to the overall decline in offspring production, although similar mechanisms involving maternal stress and provisioning underlie all three (Koski and Kuosa 1999; Holste and Peck 2006).

In line with our predictions, we observed a slight trend of increasing heat wave duration negatively affecting offspring production. Due to the experimental design, most females did not begin producing egg clutches until they were four or five days into the heat wave. It is likely that if the heat wave treatments were shifted to have a greater overlap with the period of egg clutch production that a stronger signal of an effect of heat wave duration would have been detected. Furthermore, while our data are consistent with a life history trade-off between survival and reproduction, testing for direct evidence of an energetic trade-off is beyond the scope of this study. It is possible that less energy was available for reproduction in the hotter heat wave treatment, which led to the observed decline in reproduction, rather than a re-allocation of energy away from reproduction to processes prioritizing survival.

We observed declines in reproduction within the 32°C treatment despite similar survival between the two heat wave intensity treatments. Based on summer field temperatures, we predicted that survivorship would decrease more rapidly in the 32°C treatments than in the 26°C treatments, because individuals rarely experience 32°C in the field. At Botany Bay, daily splash pool temperature reaches 26°C approximately 50% of summer days, while 32°C is reached on less than 5% of summer days. It is important to note, however, that our experimental heat waves were intended to reflect summer thermal regimes, but the copepods used for the fitness assays were collected in November. Mean daily maximum temperatures for October–November are closer to 16°C and reach 20°C less than 20% of the time. Combined with a laboratory acclimation at 20°C for 12 days prior to the experiment, seasonal acclimatization to November conditions may have led to lower thermal tolerances than would be exhibited during the summer. The higher than expected mortality rates in the 26°C groups demonstrate, however, that the experimental heat wave treatments were sufficiently stressful to study both lethal and sub-lethal effects on fitness. Moreover, while reduced thermal tolerance due to seasonal acclimatization may explain a higher than expected mortality rate, it is not sufficient to account for the similar patterns of survivorship between the 26 and 32°C temperature intensity groups.

Most mortality occurred early in the experiment before heat waves were finished. It is possible that the stress of clasping contributed to high female mortality rates. Adult males exhibit pre-copulatory mate-guarding behavior by clasping immature females to ensure that a potential mate has not been previously fertilized (Burton 1985). Males will clasp developing females anywhere in the C2–C5 stage, and the clasping phase can last anywhere from one to seven days. Pre-copulatory mate-guarding behavior is commonly observed in crustaceans, and likely evolved due to intersexual conflict over pre-copula duration (Parker 1974; Jormalainen 1998). Females consistently resist male clasping, and the mate-guarding is characterized by repeated female escapes and re-capture events (personal observations). The high activity levels needed to resist male clasping likely require large energy expenditures and also cost the female in terms of reduced feeding opportunity (Jormalainen et al. 1994; Huey and Kingsolver 2019). It is possible that the interaction between clasping stress and temperature resulted in equal mortality rates between the 26 and 32°C despite the prediction that mortality increases with thermal stress.

### Heat waves in the intertidal

Extreme heat events, heat waves, are a major source of disturbance in intertidal systems (Dethier 1984). Eurythermal organisms in the intertidal routinely experience dramatic fluctuations in temperature and are predicted to be more robust to climatic extremes than organisms from stable environments (Hoffmann et al. 2003). Organisms, however, routinely encounter temperatures close to their lethal limit, and may actually be more susceptible to increasing temperatures (Somero 2010). Rapid climate change is leading to an increase in the frequency and severity of heat waves. As such, they will likely become an even more important factor structuring intertidal communities.

We manipulated the heat wave thermal regime along two axes: daily maximum temperature and duration of consecutive days of exposure. Our heat wave manipulations, however, also include differences in ramping temperatures that are confounded with maximum temperature. The ramping speeds (6-hour ramp-up and ramp-down to and from maximum temperature) were the same between the heat wave intensity groups, however, the degree increase per hour was higher for the 32°C group. In nature, heat wave structure may vary in different but equally important ways not examined here, for example, ramping speed; length of the daily maximum temperature exposure; increasing daily minimum temperatures; differences in accumulated thermal units. The interactive effects of multiple axes of heat wave structure on fitness are not well understood. For example, mortality may increase with increasing daily maximum temperatures, or through an increase in the number of degree-warming hours, which do not necessarily entail higher daily maximum temperatures (Siegle et al. 2018). Additionally, increases in daily minimum temperatures may reduce the availability of recovery time as the daily temperature recedes following afternoon highs. This recovery time allows for the repair of cellular components, and reduction in concentration of heat shock proteins, which may have deleterious effects if they persist at a high concentration for too long (Feder and Hofmann 1999). Non-linear changes in performance with temperature given Jensen's inequality (Jensen 1906; Denny 2017) show that variation in temperature can have a large effect on performance. As such, the plethora of ways temperature variation can manifest should be investigated accordingly.

### Conclusion

Our results showed that temperature *per se*, but not the number of consecutive days of heat wave exposure, negatively impacted reproduction, but not survival, in *T. californicus*. Consequently, our study contributes to a small but growing literature on the impact of heat waves on demography, and shows that sublethal effects of heat waves may impact population vital rates even if mortality rates are comparable between thermal regimes of varying temperature intensity. Future studies should investigate how manipulating other aspects of heat wave structure, such as increasing the daily maximum temperature exposure time, affects individual survival and reproduction. Additionally, the short-term effects of sublethal heat stress will not necessarily impact population persistence over longer time scales (Foley et al. 2019). Examining the effects of transient heat stress on both short-term individual and longer-term population processes will be key to understanding the full range of heat wave effects on populations and communities.

## Supplementary Material

obac005_Supplemental_File

## Data Availability

The data is available on Dryad. Siegle, Matthew (2022), tigriopus_fitness_assays, Dryad, Dataset, https://doi.org/10.5061/dryad.gqnk98spw
