# Supplementary material for: Heat Wave Intensity Drives Sublethal Reproductive Costs in a Tidepool Copepod
Source: Integr Org Biol. 2022 Jan 31;4(1):obac005. doi: 10.1093/iob/obac005 (PMC8896982; doi:10.1093/iob/obac005)
Supplement: obac005_Supplemental_File [file obac005_supplemental_file.docx]

**Supplementary Information**

Supplementary Table 1. Daily maximum splash pool water temperatures from Botany Bay, British Columbia. The percentage of days where daily maximum temperatures reached 26°C, 30°C, or 32°C for each annual group of splash pools is shown. Daily maximum temperatures reached 26°C ~50% of days whereas temperatures reached 32°C less than 5% of the time.

| Year | # pools | Date range | # days | Freq. 26° | Freq. 30° | Freq. 32° |
| --- | --- | --- | --- | --- | --- | --- |
| 2015 | 3 | July-Sept | 82 | 34 – 55% | 0 – 11% | 0 – 5% |
| 2016 | 5 | May-Aug | 86 | 47 – 54% | 7 – 15% | 0 – 5% |

Supplementary Figure 1. Map of the *Tigriopus californicus* collection site: Botany Bay in Botanical Beach Provincial Park, BC, Canada. Map courtesy of J. Cristiani.

Supplementary Figure 2. Examples of typical daily temperature profiles for six splash pools in Botany Bay (BY), BC, from 2-June-2016 through 27-June-2016. Each panel represents one splash pool. Horizontal lines are shown at 20°C, 26°C and 32°C.
